# Chronic low back pain management: clinical and psychophysiological outcomes of multimodal approaches—a randomised controlled trial on yoga and mindfulness

**DOI:** 10.1136/bmjsem-2025-002697

**Published:** 2025-06-08

**Authors:** Yusra Saleem, Shamoon Noushad, Sadaf Ahmed, Basit Ansari

**Affiliations:** 1 Department of Health, Physical Education and Sports Sciences, University of Karachi, Karachi, Pakistan; 2 Advance Educational Institute and Research Centre, Karachi, Pakistan; 3 Psychophysiology Research Unit, Department of Physiology, University of Karachi, Karachi, Pakistan

**Keywords:** Sports & exercise medicine, Physical activity, Intervention, Exercise physiology, Quality of life

## Abstract

Chronic low back pain (CLBP) presents as a widespread medical issue which severely affects personal health status while generating substantial economic expenses. Traditional treatment methods often have limited efficacy, necessitating the exploration of alternative therapies such as yoga and mindfulness-based stress reduction (MBSR). This study aims to compare the efficacy of Sphinx pose yoga and MBSR in managing CLBP. The focus is on evaluating improvements in pain intensity, functional disability, quality of life, heart rate variability and physiological markers associated with CLBP. This multicentre parallel-arm randomised controlled trial will compare the efficacy of yoga to MBSR for CLBP in healthcare providers. Participants will be randomly assigned to one of four groups: Sphinx pose yoga therapy (Group A), MBSR (Group B), usual care (Group C) and a combined yoga-MBSR intervention (Group D). Each intervention will last 12 weeks. Primary outcomes include pain intensity, functional disability (Oswestry Questionnaire) and physiological markers (cortisol, β-endorphins, substance-P, interleukin-6, C reactive protein). Secondary outcomes encompass quality of life (WHO Quality of Life), stress (Sadaf Stress Scale), depression (Beck Depression Inventory), anxiety (Generalised Anxiety Disorder-7) and heart rate variability. Data will be collected at baseline (week 0), at the end of the intervention (week 12) and 12 weeks after the intervention (week 24). Trial registration number NCT06910982.

**WHAT IS ALREADY KNOWN ON THIS TOPIC**

- Chronic low back pain (CLBP) is a widespread condition that impairs quality of life and contributes significantly to socioeconomic costs.
- Healthcare providers are particularly vulnerable to CLBP due to the physical and emotional demands of their profession.
- Conventional treatments often have limited effectiveness, leading to growing interest in alternative therapies like yoga and mindfulness-based stress reduction (MBSR).
- Yoga and MBSR have shown promise individually for managing chronic pain, but their specific comparative effects on CLBP, particularly in healthcare providers, are not well established.

**WHAT THIS STUDY ADDS**

- This study is among the first to systematically compare yoga, MBSR and their combination against usual care in a randomised controlled multicentre trial specifically among healthcare providers with CLBP.
- It uniquely integrates clinical (pain, disability, quality of life, mental health) and psychophysiological outcomes (inflammatory biomarkers and heart rate variability) to offer a comprehensive evaluation of interventions.
- By focusing on a specific yoga posture (Sphinx pose) and structured mindfulness practice, it provides nuanced insights into targeted non-pharmacological treatments for CLBP.

**HOW THIS STUDY MIGHT AFFECT RESEARCH, PRACTICE OR POLICY**

- Results could establish evidence-based, non-pharmacological treatment protocols for managing CLBP among healthcare workers, a high-risk population.
- It may encourage broader integration of mind-body interventions like yoga and MBSR into clinical guidelines for chronic pain management.
- The use of physiological markers and heart rate variability could influence future research designs, encouraging the adoption of more objective biomarkers alongside self-reported outcomes.
- Findings may support policy shifts toward preventive care strategies in occupational health settings, reducing the burden of CLBP-related disability.

## Introduction

 Chronic low back pain (CLBP) is a prevalent global health issue with varying prevalence rates across different populations. In Turkey, the lifetime prevalence of CLBP was reported as 13.1%.^1^ A study in Northern India found a prevalence of 16.1% among adults.^2^ In KwaZulu-Natal, South Africa, the prevalence was 18.1%, with women having a higher rate than men.^3^ In Benin, the overall prevalence was 35.5%, with rural areas (40.2%) showing higher rates than urban areas (30.68%).^4^ Common risk factors for CLBP include increasing age, being underweight or overweight, lack of physical activity, smoking, alcohol consumption, manual work and poor posture.^2^,^4^ Other associated factors include education level, marital status and working status.^4^

CLBP has complex pathological mechanisms involving peripheral and central systems, with inflammation playing a crucial role.^5^ Studies have shown elevated levels of pro-inflammatory biomarkers in patients with CLBP, including C reactive protein (CRP), interleukin-6 (IL-6) and tumour necrosis factor-α.^6 7^ These inflammatory markers are associated with increased pain intensity and central sensitisation.^5^ However, the inflammatory profiles may differ based on racial background, with non-Hispanic black and non-Hispanic white individuals showing distinct patterns of biomarker associations with pain outcomes.^6^ Anti-inflammatory markers like IL-4 and IL-10 have also been found to have inverse relationships with CLBP symptoms.^6 7^ These findings highlight the importance of considering inflammatory biomarkers in understanding CLBP pathophysiology and developing targeted treatments.

Recent studies have explored the relationship between CLBP and heart rate variability (HRV). Evidence suggests that patients with CLBP exhibit reduced HRV and sympathetic predominance compared with healthy controls.^8^ During passive viewing of daily activity photographs, patients with CLBP showed significant changes in HRV frequency domains, increased pain sensitivity and higher pain intensity.^8^ In older adults with CLBP, emotional self-efficacy was found to moderate the relationship between low-frequency HRV and physical functioning.^9^ Interestingly, a comparison between chronic neck pain and patients with CLBP revealed insignificant differences in autonomic dysfunction.^10^ However, disability and self-efficacy correlated with HRV in patients with neck pain, while catastrophising and kinesiophobia showed stronger correlations in patients with CLBP. These findings highlight the complex interplay between chronic pain, autonomic regulation and psychological factors, suggesting potential avenues for intervention and further research.

Conventional therapies include pharmacological and non-pharmacological approaches, but their effectiveness is not always optimal.^11^ Recent guidelines recommend a multimodal management approach, combining non-pharmacological treatments such as exercise therapy, physical activity and education with pharmacological interventions in selected cases.^12^ Experts have found that yoga functions as a successful treatment method for CLBP, although it extends its benefits above classic physical exercise methods. Yoga demonstrates its ability to decrease pain along with disability and medication needs while boosting spinal flexibility and quality of life for patients with CLBP according to research.^13^ Yoga’s comprehensive approach, incorporating postures, breath regulation, mindfulness and meditation, may contribute to its effectiveness.^13 14^ Furthermore, mindfulness-based stress reduction (MBSR) and other meditation-based therapies are also found effective in managing CLBP. A meta-analysis found that MBSR significantly improved physical function in patients with CLBP at 8 weeks and 6 months follow-up.^15 16^

Building on our previously published research demonstrating the effectiveness of Sphinx pose yoga in managing CLBP, we have now designed a multicentre randomised controlled trial to compare the efficacy of Sphinx pose yoga with MBSR in healthcare providers (HCPs) with CLBP. This study will assess outcomes including pain intensity, functional disability, inflammatory biomarkers and HRV, aiming to generate robust evidence for optimising non-pharmacological interventions and improving the quality of life in individuals affected by CLBP.

## Methodology

### Study design

This multicentre parallel-arm randomised controlled trial will compare the efficacy of yoga to MBSR for CLBP. The study will have four parallel arms: Group A (yoga therapy), Group B (MBSR), Group C (usual care) and Group D (combined yoga and MBSR). Participants will be randomised into these groups and receive the respective interventions for 12 weeks.

### Participants

HCPs with CLBP will be recruited from various healthcare facilities.

### Sample size calculation

**Figure 1:**
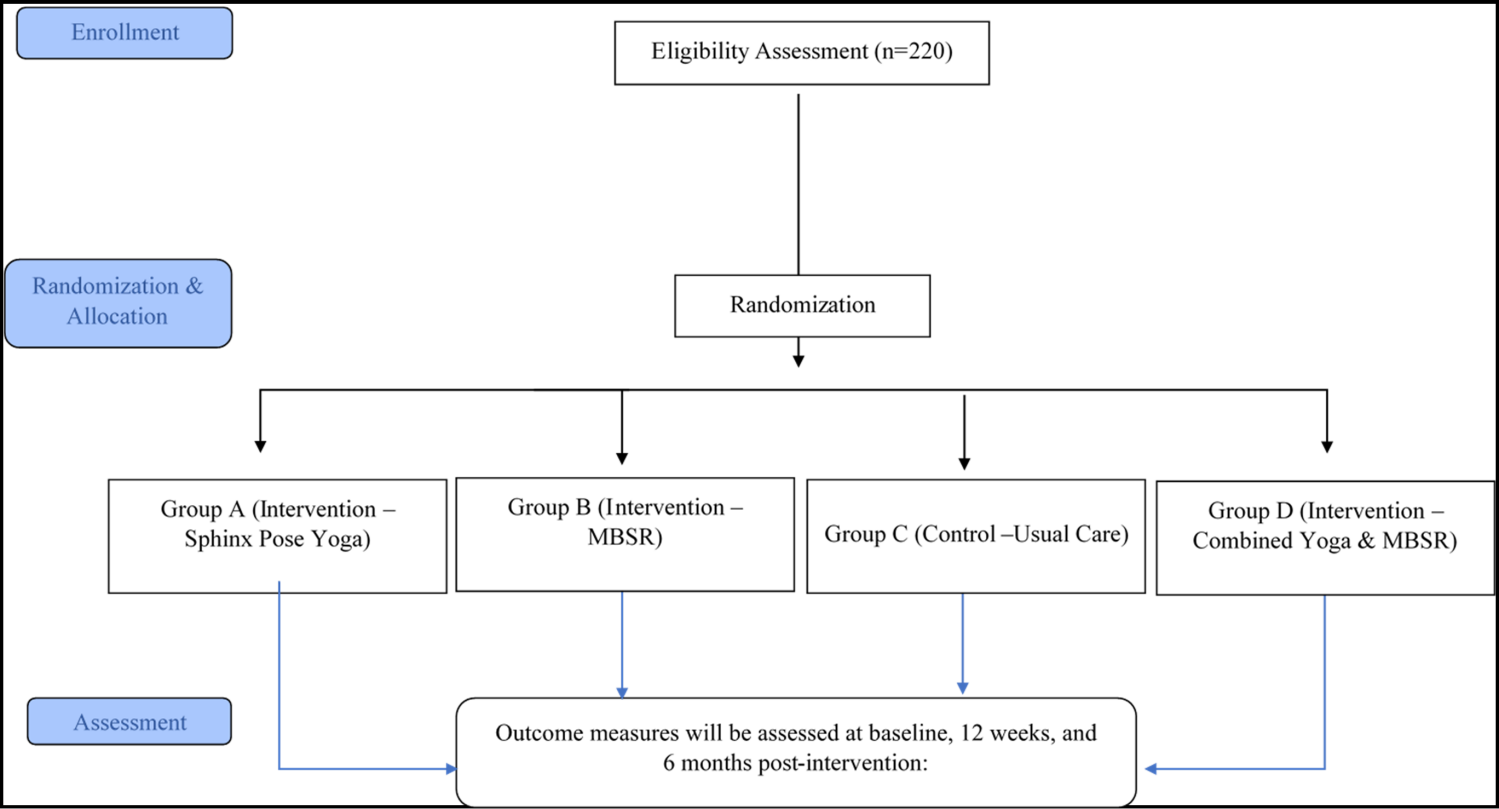
Study flow. MBSR, mindfulness-based stress reduction.

The sample size calculation is based on findings from our prior randomised controlled trial evaluating Sphinx pose yoga therapy in healthcare professionals with CLBP.^17^ In that study, participants in the yoga group showed a significant improvement in functional disability, with a mean reduction of 4.4 points on the Oswestry Disability Index (ODI) and an SD of 3.69, corresponding to an effect size (Cohen’s d) of approximately 1.2. For the current study, we have conservatively assumed a smaller effect size of 0.6 to ensure adequate power even if the true effect is more modest. This value is consistent with effect sizes commonly reported in the literature on mind-body interventions such as yoga and MBSR for CLBP. Using a significance level of 0.05 and a power of 0.80, the required sample size is 44 participants per group. To account for a potential 20% dropout rate, the final sample size has been increased to 55 participants per group, totalling 220 participants across the four study groups (figure 1).

### Randomisation

Randomisation will be conducted using computer-generated sequences, stratified by key demographics (eg, age, gender).

### Blinding

Although participants and intervention providers cannot be blinded due to the nature of the interventions, all outcome assessors will be blinded to group allocation. Assessors will not be involved in the intervention delivery and will collect and score outcome measures without knowledge of the participant’s group assignment. This is especially important given the use of subjective outcomes such as pain intensity and disability.

### Eligibility criteria

#### Inclusion criteria

- Both genders between 25 and 45 years of age.
- Subjects having low back pain complaints and have visited the healthcare provider in recent days.
- Numerical Pain Rating (NPR) scale score ≥2 for their pain intensity.
- Roland Morris Disability Questionnaire subject’s score should be ≥4.
- Fear Avoidance Beliefs Questionnaire work subscale score must be <19.

#### Exclusion criteria

- Subjects with high risk for physical injuries during exercise.
- Pregnant and/or lactating women.
- Subjects with musculoskeletal disorders.
- Medical contraindications to physical activity or yoga (eg, severe cardiovascular or neurological conditions).
- Current psychiatric illness or history of severe depression or anxiety, defined as:
  - Beck Depression Inventory-II (BDI-II) score >29 (severe depression), and/or
  - Generalised Anxiety Disorder-7 (GAD-7) score >15 (severe anxiety).
- Ongoing use of psychoactive medications or enrolment in other structured psychological interventions.

### Interventions

Participants will be randomly assigned to one of four intervention groups (see table 1).

**Table 1:** Detailed intervention protocols and session schedules across study groups

Group	Intervention	Description	Duration/frequencies/sessions
Group A	Sphinx pose yoga therapy	Participants will engage in Sphinx pose yoga therapy, focusing on back extension poses. The programme follows a simplified protocol aimed at improving accessibility, adherence and safety, especially for high-risk occupational groups.^17^ Sessions include warm-up stretching, targeted back extension and cool-down, with a focus on minimalism to ensure clinical benefit.	Frequency: 3 times per week for 12 weeks
			Session duration: 40 min per session
			Total sessions: 36 sessionsTotal contact time: 24 hours
Group B	MBSR (mindfulness-based stress reduction)	Participants will engage in a structured MBSR programme, including mindfulness meditation, body scan exercises, gentle yoga-based movements and group discussions. The programme follows the standard 8-week MBSR curriculum, extended to 12 weeks to match the duration of other interventions.^31^ Participants will be encouraged to practise daily at home using guided recordings.	Frequency: 1 session per week for 12 weeks
			Session duration: 2 hours per session (weeks 1–8), 1 hour per session (weeks 9–12)
			Total sessions: 12 sessionsTotal contact time: 20 hours
Group C	Usual care	Participants will receive guidance based on The Back Book, which includes advice on remaining active, avoiding prolonged rest and using simple pain control methods like over-the-counter analgesics. The approach encourages low-impact exercises (eg, walking, swimming) and basic stress reduction strategies, such as relaxation and breathing techniques.	Frequency: As per individual need, based on standard care guidelines
			Duration: 12 weeks
			Total sessions: Minimal contact, no structured sessions
Group D	Combined yoga and MBSR	Participants in Group D will receive a comprehensive, multimodal intervention consisting of full-dose Sphinx pose yoga therapy and a standardised mindfulness-based stress reduction (MBSR) programme. This design aims to evaluate the additive or synergistic benefits of combining two evidence-based approaches targeting both physical and psychological dimensions of chronic low back pain without compromising the integrity of either protocol. Sessions will be scheduled on non-consecutive days to minimise participant fatigue and optimise engagement.	Frequency: Yoga: three times/week MBSR: once a week Duration: Yoga: same as Group A MBSR: same as Group B Total sessions: 36+12=48Total contact time: 24+20=44 hours

### Outcome measures

Outcome measures will be assessed at baseline (week 0), at the end of the intervention (week 12) and 12 weeks after the intervention (week 24).

#### Primary outcomes

- NPR Scale: The NPR Scale is a widely used tool for assessing pain intensity.
- Oswestry Low Back Pain Disability Questionnaire^18^: ODI is considered a standard to assess low back functionality.
- Physiological markers: Cortisol, β-endorphins, substance-P, IL-6 and CRP.

#### Secondary outcomes

- WHO Quality of Life (WHOQOL)^19^: WHOQOL measures the quality of life.
- Sadaf Stress Scale (SSS)^20^: The physical stress scale subsection of the SSS (reviewed version) will be used to assess the degree of stress in relation to physical activities and its contribution to CLBP.
- BDI^21^: The BDI is a questionnaire used to assess the severity of depression symptoms.
- GAD-7 scale^22^: The GAD-7 is a brief screening tool used to assess the severity of generalised anxiety disorder symptoms.
- HRV: HRV, measured through wearable devices, reflects the variation in time intervals between consecutive heartbeats and will be measured using the Alive Biofeedback and Neurofeedback Device GP8.

### Biochemical assessment

Blood samples for biomarkers (cortisol, β-endorphins, substance-P, IL-6, CRP) will be collected at three points: baseline (week 0), post-intervention (week 12) and follow-up (week 24), between 08:00 and 09:00 in a fasting state to minimise diurnal and metabolic variability. Participants will be instructed to avoid vigorous physical activity, caffeine and analgesics 12–24 hours before sampling. The collected samples will be centrifuged within 30 min and stored at −80°C. All biomarkers will be analysed using standardised ELISA kits following international protocols.

### Study timeline

- Baseline assessment and participant enrolment (week 0).
- Intervention delivery (1–12 weeks).
- First follow-up (at the end of the intervention—week 12).
- Second follow-up (12 weeks after the intervention—week 24).

### Statistical analysis

Data will be analysed using mixed-effects models, incorporating fixed effects (intervention group) and random effects (participant-level variability). Stratification variables (age and gender) will be included as covariates to adjust for stratified randomisation. Additional analyses will include χ^2^ tests for categorical variables, analysis of variance (ANOVA) for continuous variables and linear regression models to explore associations between clinical outcomes and potential predictors. All analyses will be performed on an intention-to-treat basis.

### Adverse events monitoring

Given the nature of the interventions (yoga and mindfulness-based practices), we anticipate minimal risk. However, we recognise the potential for minor adverse effects, such as muscle soreness, fatigue or temporary emotional discomfort. To safeguard participants, adverse events will be monitored throughout the study. At each session, participants will be asked to report any adverse experiences or discomforts. Serious adverse events, if they occur, will be reported immediately to the study’s ethics committee and addressed by the study team.

An adverse event log will be maintained, with a clear process for assessment, documentation and management of any adverse events. Participants will be informed of whom to contact should they experience any unexpected symptoms or issues related to the interventions. If an adverse event occurs, appropriate medical intervention will be sought, and the participant may be withdrawn from the study if necessary to ensure their health and safety.

## Discussion

CLBP is a growing epidemic, particularly among HCPs, with prevalence estimates for nurses ranging from 50% to 80%.^23^ The biopsychosocial model of chronic pain highlights the complex interplay of biological, psychological and social factors in CLBP.^24^ Conventional treatments have shown limited effectiveness, with less than 50% of patients experiencing relief after 1 year.^25^ Non-pharmacological therapies like physical therapy, yoga and acupuncture offer moderate benefits at best.^26^ While opioids have been a common treatment, recent guidelines recommend limiting their use due to safety concerns.^26^ Novel approaches being explored include chemonucleolysis, platelet-rich plasma injections and stem cell therapies.^18^ Additionally, mind-body exercises such as yoga and tai chi show promise in addressing both physiological and psychological aspects of CLBP.^23^

Mindfulness-based interventions (MBIs) and yoga have shown promise in managing chronic pain. Studies indicate that these practices can reduce pain perception, improve mobility and enhance overall well-being.^27^ A systematic review of MBSR for CLBP revealed significant improvements in pain measures, quality of life and mental health compared with control groups.^28^ An experimental single-case study on meditation-based lifestyle modification, a yoga-based intervention, demonstrated positive effects on pain intensity, quality of life and pain self-efficacy in patients with chronic pain.^29^ Additionally, a review of yoga and other meditative movement therapies confirmed their efficacy in reducing chronic pain symptoms, emphasising the importance of professional instruction.^30^ These findings suggest that MBIs and yoga can be valuable components of multidisciplinary pain management strategies, potentially reducing reliance on pain medications. Building on this evidence, our previous randomised controlled trial demonstrated that yoga significantly improved psychophysiological biomarkers among healthcare professionals with CLBP.^17^ These results provide a strong foundation for the current study’s expanded scope.

This study is distinctive in its integration of both subjective and objective outcome measures to evaluate the efficacy of Sphinx pose yoga and MBSR in managing CLBP. Alongside traditional self-reported assessments of pain intensity, functional disability, quality of life, depression, anxiety and stress, the trial incorporates objective physiological markers such as cortisol, β-endorphins, substance-P, IL-6, CRP and HRV. By combining clinical and psychophysiological data, this approach provides a more comprehensive understanding of the mechanisms through which mind-body interventions influence chronic pain and overall well-being.

## Data Availability

Data are available upon reasonable request.
